# Comorbidities and Clinical Overlap of Cluster Headache with Other Primary Headache Disorders: A Narrative Review

**DOI:** 10.3390/diagnostics16162498

**Published:** 2026-08-07

**Authors:** Shusheng Jiao, Jianxia Zhi

**Affiliations:** Department of Neurology, Bethune International Peace Hospital, Shijiazhuang 050082, China; xiaojiao5678@sina.com

**Keywords:** cluster headache (CH), trigeminal autonomic cephalalgias (TACs), primary headache, comorbidity, overlap, narrative review

## Abstract

Cluster headache (CH) is recognized as one of the most severe primary headache disorders and belongs to the group of trigeminal autonomic cephalalgias (TACs). Traditionally regarded as a distinct clinical entity under the International Classification of Headache Disorders, 3rd edition (ICHD-3), CH has been increasingly documented over the past two decades to frequently co-occur or overlap with other primary headache disorders, challenging this conventional view. This narrative review synthesizes available evidence from the PubMed and ScienceDirect databases on the comorbidity or clinical overlap between CH and other primary headache disorders. It focuses primarily on the co-occurrence of CH with migraine, tension-type headache, other TACs, primary stabbing headache, and medication-overuse headache. In addition, it explores potential clinical overlaps between CH and several other primary headaches, including cough headache, headache associated with sexual activity, thunderclap headache, and external-pressure headache. This review highlights that comorbidity of CH with other primary headache disorders is clinically significant yet frequently underrecognized, underscoring an urgent need for systematic prospective investigations to improve diagnostic accuracy and treatment outcomes.

## 1. Introduction

Cluster headache (CH) is widely recognised as one of the most severe primary headache disorders, belonging to the group of trigeminal autonomic cephalalgias (TACs). Traditionally, CH has been viewed as a distinct clinical entity with well-defined diagnostic criteria under the International Classification of Headache Disorders, 3rd edition (ICHD-3). However, accumulating clinical observations over the past two decades have challenged this simplistic view [1]. A growing body of evidence suggests that CH frequently co-occurs with other primary headache disorders [2,3]. Patients with CH may simultaneously meet the diagnostic criteria for migraine, tension-type headache (TTH), or primary stabbing headache (PSH), among others [4,5]. These overlapping phenotypes are not merely coincidental but may reflect shared pathophysiological mechanisms, genetic susceptibilities, or iatrogenic modifications of the clinical course, such as medication-overuse headache (MOH) [6].

The advent of the ICHD-3 has facilitated more rigorous phenotyping of headache disorders, thereby allowing clinicians and researchers to identify and characterise comorbid presentations with greater precision. This narrative review aims to synthesise the available evidence from PubMed and ScienceDirect databases regarding the comorbidity or overlap of CH with other primary headache disorders. Specifically, we will discuss the clinical patterns, proposed pathophysiological links, and therapeutic implications of each commonly observed comorbidity. By doing so, we hope to provide a clinically useful resource for headache specialists and to highlight important gaps in knowledge that merit future investigation.

## 2. Comorbidity or Overlap of CH with Other Primary Headache Disorders

**Figure 1:**
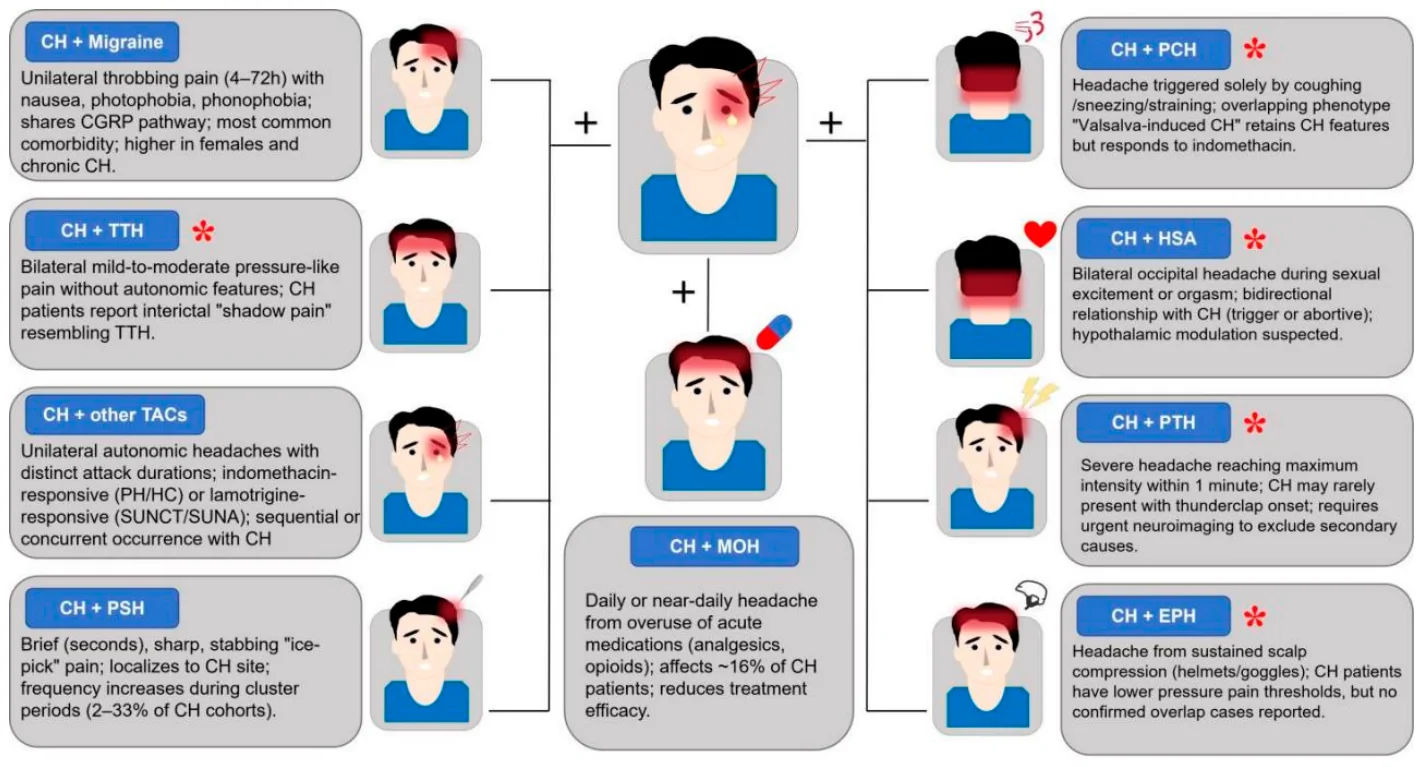
Overview of comorbidities and clinical overlap between cluster headache and other primary headache disorders. Cluster headache (CH) is positioned at the center. * indicates possible overlaps that require further confirmation due to a lack of well-documented literature evidence. Abbreviations: TTH, tension-type headache; TACs, trigeminal autonomic cephalalgias, including paroxysmal hemicrania (PH), hemicrania continua (HC), short-lasting unilateral neuralgiform headache attacks with conjunctival injection and tearing (SUNCT), and short-lasting unilateral neuralgiform headache attacks with cranial autonomic symptoms (SUNA); PSH, primary stabbing headache; MOH, medication-overuse headache; PCH, primary cough headache; HSA, headache associated with sexual activity; PTH, primary thunderclap headache; EPH, external-pressure headache.

**Figure 2:**
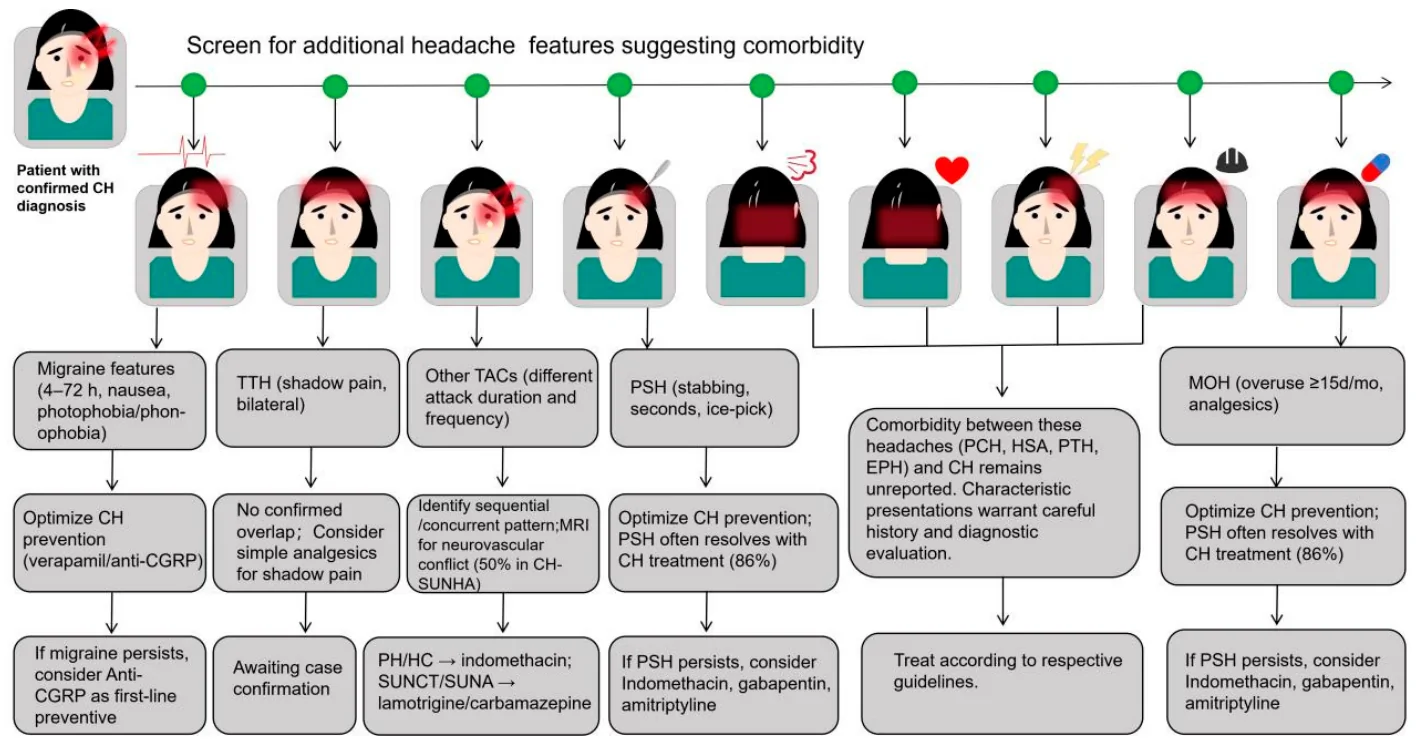
Clinical algorithm for managing cluster headache (CH) based on documented comorbidities and clinical overlaps. This algorithm provides a stepwise approach to patient assessment and treatment when CH co-occurs with other headache disorders. Abbreviations: PSH, primary stabbing headache; MOH, medication-overuse headache; HSA, headache associated with sexual activity; TTH, tension-type headache; EPH, external-pressure headache; TACs, trigeminal autonomic cephalalgias (PH, paroxysmal hemicrania; HC, hemicrania continua; SUNCT, short-lasting unilateral neuralgiform headache attacks with conjunctival injection and tearing; SUNA, short-lasting unilateral neuralgiform headache attacks with cranial autonomic symptoms).

Based on a comprehensive synthesis of available literature from PubMed and ScienceDirect databases, this review identifies eight distinct headache disorders that have been documented to coexist with or share clinical features with CH. Although MOH is formally classified as a secondary headache under ICHD-3, it is discussed here because it inevitably arises from a primary headache and substantially modifies the clinical course and treatment response of CH. The headache disorders covered in this review include migraine, TTH, other TACs (including paroxysmal hemicrania [PH], hemicrania continua [HC], short-lasting unilateral neuralgiform headache attacks with conjunctival injection and tearing [SUNCT], and short-lasting unilateral neuralgiform headache attacks with cranial autonomic symptoms [SUNA]), along with PSH, MOH, primary cough headache (PCH), headache associated with sexual activity (HSA), primary thunderclap headache (PTH), and external-pressure headache (EPH). Figure 1 provides a visual overview of these comorbidities and clinical overlaps, and Figure 2 describes a clinical algorithm for managing CH based on documented comorbidities and clinical overlaps. To facilitate clinical recognition of these comorbidities, we have summarized the reported prevalence rates of each primary headache disorder co-occurring with CH in Table 1. The following sections discuss each of these disorders in detail, focusing on their epidemiological characteristics, pathophysiological mechanisms, and therapeutic implications when co-occurring with CH.

### 2.1. Comorbidity of CH with Migraine

CH and migraine are two of the most debilitating primary headache disorders, imposing a substantial global burden on affected individuals and healthcare systems [1,3]. While clinically distinguishable according to ICHD-3, these two conditions share prominent features including unilateral pain, cranial autonomic symptoms, and response to certain therapeutic agents such as triptans [10]. The comorbidity between CH and migraine is increasingly recognized, with clinical observations suggesting that a subset of patients exhibits overlapping phenotypes that challenge traditional diagnostic boundaries. Large-scale epidemiological studies have demonstrated that migraine is among the most common comorbid conditions in patients with CH [11]. Beyond simple comorbidity—the co-occurrence of two independently diagnosable disorders—a subset of patients presents with overlapping clinical features that do not cleanly fit into either diagnostic category. Terms such as “cluster-migraine,” “migraine-cluster,” “cyclical migraine,” and “clustering episodes of migraine” have been used to describe these intermediate phenotypes [12]. Whether these represent true comorbid states, diagnostic uncertainty, distinct nosological entities, or manifestations of shared underlying pathophysiology remains a matter of ongoing debate [3]. Moreover, case reports of rare phenotypes, such as hemiplegic cluster headache—in which typical CH attacks are accompanied by visual, sensory, and/or aphasic migrainous auras that may evolve into reversible hemimotor symptoms [13]. While not formally included in the ICHD-3, such cases have been sporadically reported since their first description in 2002, raising questions about shared pathophysiological mechanisms that can produce hybrid phenotypes.

Epidemiology of CH-migraine comorbidity. Epidemiological studies consistently indicate that migraine is a common comorbidity in patients with CH, although reported prevalence rates vary depending on study methodology, population characteristics, and diagnostic criteria employed. A large-scale retrospective analysis using the Epic Cosmos electronic health record database, which included 152,727 patients with CH across multiple healthcare systems in the United States, identified migraine as one of the most frequent comorbid conditions among patients with CH [11]. This study, representing one of the largest cohorts of CH patients analyzed to date, underscores the substantial clinical overlap between these two disorders. Data from the Korean Cluster Headache Registry, involving 16 tertiary hospitals over a five-year period (2016–2021), provided additional insights into regional variations [14]. This multicenter study found that female patients with CH demonstrated higher comorbidity with migraine compared to males, and that women with CH more frequently developed the chronic form of the disorder. These sex-specific differences suggest potential hormonal or genetic influences on the expression of both conditions. The clinical significance of this comorbidity extends beyond mere numerical association. Patients with comorbid CH and migraine experience greater overall headache burden, with studies reporting significantly impaired quality of life and elevated suicidality risk compared to patients with either disorder alone [14]. This highlights the importance of recognizing and appropriately managing both conditions when they co-occur.

Pathophysiological mechanisms. The pathophysiological relationship between CH and migraine is characterized by both shared mechanisms and distinct features. The trigeminovascular system represents a common pathway for both disorders, wherein activation leads to the release of calcitonin gene-related peptide (CGRP), a central molecular mediator implicated in acute attacks of both CH and migraine [1,10]. Elevated CGRP levels during active phases and the efficacy of anti-CGRP monoclonal antibodies underscore this shared mechanism. A recent meta-analysis by Silva et al. (2025), pooling 504 patients from 6 studies, reported an overall efficacy rate of 76.0% for galcanezumab in reducing CH attack frequency [15]. In episodic CH, the pivotal Phase 3 RCT demonstrated significant efficacy (Goadsby et al., 2019), while in chronic CH, although the RCT did not meet its primary endpoint [16], real-world studies have shown promising response rates of approximately 50–65% in refractory patients [17,18]. Safety data from the meta-analysis showed treatment-emergent adverse events in 48.0% of patients, predominantly mild and dose-dependent, with fewer events at the 240 mg dose compared to 300 mg (28.0% vs. 80.0%). Together, these findings support the role of CGRP pathway modulation as a shared therapeutic target across the CH–migraine comorbidity spectrum. However, neuroimaging reveals a divergent role of the hypothalamus: while CH is marked by consistent hypothalamic hyperactivity correlating with its circadian and circannual rhythmicity, migraine involves less pronounced hypothalamic changes and instead features cortical and brainstem (especially dorsal pons and periaqueductal gray) involvement [1,19]. Both disorders exhibit central sensitization on quantitative sensory testing, yet migraine demonstrates a characteristic lack of habituation to repeated stimuli across multiple sensory modalities, whereas in CH, this lack of habituation is lateralized to the affected side during active phases [10,20]. Genetically, both show familial aggregation—first-degree relatives of CH patients have a 5- to 15-fold increased risk, and those of migraine patients a ~3-fold increased risk—but CH displays stronger heritability with possible autosomal dominant inheritance, whereas migraine is polygenic with numerous susceptibility loci [21,22]. These overlapping and divergent pathophysiological features provide a framework for understanding the comorbidity between CH and migraine.

Therapeutic implications. From a therapeutic perspective, the management of patients with comorbid CH and migraine requires an integrated approach that addresses both conditions. For acute treatment, subcutaneous sumatriptan (6 mg) remains the gold standard for CH attacks due to its rapid relief within 10–15 min, while oral triptans are typically sufficient for migraine attacks, though subcutaneous administration may be reserved for severe episodes [23,24]. High-flow oxygen (100% at 12–15 L/min for 15–20 min) is uniquely effective for CH but not for migraine, and a dramatic response to oxygen should raise clinical suspicion of a CH component in comorbid patients [25]. In terms of preventive therapy, traditional strategies diverge: verapamil is first-line for CH, whereas beta-blockers, antiepileptics (topiramate, valproate), and tricyclic antidepressants are commonly used for migraine [19,26]. However, the advent of anti-CGRP monoclonal antibodies, particularly galcanezumab, has provided a shared therapeutic option, with real-world studies demonstrating over 60% of CH patients achieving at least a 50% reduction in attack frequency, and similar efficacy in migraine [27,28,29]. For patients with confirmed comorbidity, a stepped approach is recommended: acute attacks may be treated with subcutaneous sumatriptan (for CH-type episodes) or oral triptans (for milder migraine attacks), a trial of high-flow oxygen can help confirm the presence of a CH component, anti-CGRP monoclonal antibodies should be considered as first-line preventive therapy to address both disorders simultaneously, and neuromodulation techniques (e.g., non-invasive vagus nerve stimulation, sphenopalatine ganglion stimulation) may serve as adjunctive or rescue options for both conditions [30,31]. Finally, clinicians must remain vigilant for medication-overuse headache, which can develop in comorbid patients who overuse acute medications and may complicate both the clinical presentation and treatment response.

### 2.2. Possible Comorbidity of CH with Tension-Type Headache (TTH)

CH and TTH represent two distinct primary headache disorders with markedly different clinical profiles. CH is characterized by severe, strictly unilateral pain accompanied by ipsilateral cranial autonomic symptoms and a distinct circannual and circadian rhythmicity, whereas TTH typically presents with bilateral, mild-to-moderate pressure-like pain without autonomic features and is the most prevalent primary headache disorder worldwide. Marmura and Young (2012) reviewed the phenomenon of interictal pain in primary headache syndromes and noted that patients with trigeminal autonomic cephalalgias, including CH, commonly experience milder interictal pain that is typically unilateral and localized to the side of attacks [32]. This interictal pain is generally less severe than acute CH attacks and may clinically resemble TTH in its quality and intensity. In a 2025 case series of 10 patients with coexisting CH and SUNHA, Jansen and Mulleners documented that several patients experienced “shadow pain” during interictal periods. This pain was located either in the region of the CH attacks or bilaterally in the forehead [2]. The authors’ use of the term “shadow pain” reflects a clinical reality that CH patients do experience bilateral milder pain between attacks, suggesting the possible presence of comorbid TTH during interictal periods. However, to date, no peer-reviewed case reports or clinical studies have explicitly documented a confirmed comorbidity between CH and TTH, nor have they systematically characterized interictal pain in CH patients as meeting formal TTH diagnostic criteria. Given the well-recognized low healthcare-seeking behavior and low reporting rates for TTH in real-world clinical practice—where many individuals with TTH never consult a physician—it is plausible that comorbid CH-TTH is underdiagnosed. Specifically, the mild interictal pain reported by CH patients could be misattributed to residual CH symptoms rather than recognized as a distinct TTH phenotype. Future prospective studies employing systematic diagnostic interviews and headache diaries are warranted to clarify the prevalence, clinical characteristics, and pathophysiological relationship between interictal pain in CH and TTH, and to determine whether such presentations represent true comorbidity, a shared biological spectrum, or merely a nonspecific pain residual.

### 2.3. Comorbidity of CH with Other TAC Disorders (TACs)

CH is the most common of TACs, a group of primary headache disorders characterized by unilateral head pain accompanied by ipsilateral cranial autonomic features [11]. The TACs encompass CH, PH, HC, and SUNCT/SUNA [33]. While these disorders are typically considered distinct entities defined by specific attack durations, frequencies, and therapeutic responses, accumulating evidence suggests that different TACs may co-occur within the same patient, either concurrently or sequentially over time [33]. This phenomenon challenges traditional diagnostic boundaries and supports the hypothesis of shared pathophysiological mechanisms across the TACs spectrum.

Epidemiology of CH-TACs comorbidity. Epidemiological data on the comorbidity between CH and other TACs remain limited, primarily due to the rarity of these disorders, and formal comorbidity rates have not been systematically established. Nevertheless, published case reports and case series provide compelling evidence that different TACs can co-occur in the same patient, either sequentially or concurrently. For instance, one documented case described the sequential occurrence of ipsilateral CH, SUNCT, and HC in a single patient over a four-year period [34]. In another case report, Centonze and colleagues documented a 42-year-old man who experienced concomitant attacks of CH and chronic paroxysmal hemicrania (CPH) [35]. Lisotto et al. reported the first case of two different contralateral TACs co-existing in the same patient—a 32-year-old man with an eight-year history of daily right-sided headaches (later diagnosed as HC) and a four-year history of episodic left-sided pain attacks consistent with episodic CH [36]. Furthermore, a case series of 10 patients with coexisting CH and short-lasting unilateral neuralgiform headache attacks (SUNHA, including SUNCT and SUNA) revealed that most cases were consecutive, with CH preceding SUNHA in seven patients by an average of 5.3 years, while the remaining two patients exhibited simultaneous onset [2]. Although the rarity of reported cases suggests that CH-TAC comorbidity is uncommon, diagnostic challenges—such as overlapping clinical features and the need for multiple therapeutic trials—may contribute to its underrecognition. Prospective registry studies are therefore warranted to establish precise prevalence estimates.

Pathophysiological mechanisms. The co-occurrence of CH with other TACs is best explained by shared pathophysiological mechanisms involving the trigeminovascular system, the trigeminal-autonomic reflex, and hypothalamic dysfunction. Evidence from a retrospective case series of 10 patients diagnosed with both CH and SUNHA provides further insight into this overlap. In this series, four patients (50%) exhibited ipsilateral trigeminal neurovascular conflict on magnetic resonance imaging (MRI) [2]. This finding suggests that trigeminal root demyelination or mechanical compression may contribute to the pathophysiology of co-occurring CH and SUNHA, potentially interacting with central mechanisms such as hypothalamic dysfunction to produce overlapping phenotypic features.

Therapeutic Implications. The management of patients with comorbid CH and other TACs is challenging because each TAC has a distinct acute and preventive treatment profile. CH attacks typically respond to subcutaneous sumatriptan (6 mg) and high-flow oxygen, whereas paroxysmal hemicrania and hemicrania continua are uniquely responsive to indomethacin, and SUNCT/SUNA often respond to lamotrigine or carbamazepine [11]. In cases of sequential TAC transformation, treatment must be adjusted dynamically: a patient initially diagnosed with CH may benefit from verapamil, but if the phenotype shifts to SUNCT, lamotrigine becomes necessary; subsequent transformation to HC requires an indomethacin trial [34]. For patients with concurrent TAC phenotypes, a combined or stepped pharmacologic approach may be required. Neuromodulation techniques, including posterior hypothalamic deep brain stimulation and non-invasive vagus nerve stimulation, have shown efficacy across multiple TACs and may offer treatment options for refractory comorbid cases [31]. When trigeminal neurovascular conflict is identified on MRI, microvascular decompression has been reported to benefit both CH and SUNHA in some patients [2]. Future prospective studies are needed to establish evidence-based guidelines for managing CH when it co-occurs with other TACs.

### 2.4. Comorbidity of CH with Primary Stabbing Headache (PSH)

PSH, also known as “ice-pick headache” or “jabs and jolts syndrome”, is a relatively common but often underrecognized primary headache disorder [37]. It is characterized by brief, sharp, stabbing pains that last from a few seconds to up to 10 s, typically occurring as single episodes or in series of several attacks per day [38]. Although CH and PSH are recognized as two distinct primary headache disorders under the ICHD-3 classification, the comorbidity and phenotypic overlap between them have been well documented. Reported comorbidity rates range from 2% to 33% for PSH in CH cohorts, and approximately 10% for CH in PSH populations [4].

Epidemiology of CH-PSH comorbidity. Population-based studies estimate the lifetime prevalence of PSH at approximately 35.2% [37], making it considerably more common than CH (0.1%). Within CH cohorts, PSH prevalence ranges from 2% to 33%, with significant variability based on diagnostic rigor. Conversely, when PSH is the index disorder, CH appears in only 10% of cases. The strong female predominance of PSH (82% in tertiary cohorts) contrasts with the male predominance of CH, suggesting that while the disorders co-occur, their demographic profiles differ [4]. In affected patients, PSH stabs are typically localized to the exact V1 site of CH pain, and their frequency increases during cluster periods and remits with CH, indicating temporal coupling.

Potential pathophysiological mechanisms. The co-occurrence of CH and PSH is best explained by shared trigeminovascular hyperexcitability. Both disorders involve spontaneous activation of nociceptive nerve fibres originating from the trigeminal and occipital nerves, and the high comorbidity rate (up to 33% in some CH series) suggests that PSH may represent an interictal expression of an underlying CH biology rather than a distinct independent disorder [4]. Functional neuroimaging studies have demonstrated hypothalamic activation in CH, while the brainstem nuclei (raphe and locus coeruleus) and cortical hyperexcitability are implicated in generating cortical spreading depression [39]. CGRP, a key mediator in trigeminal autonomic cephalalgias, is also involved in neurogenic inflammation that may facilitate both CH attacks and stabbing paroxysms [40]. The observation that PSH frequently occurs during ongoing CH attacks (69% in one cohort) supports a shared permissive biological state rather than independent pathological processes [4].

Therapeutic implications. Management of CH-PSH comorbidity should prioritise treatment of the underlying CH, as effective CH preventive therapy often improves stabbing pains. Among patients receiving treatment for their primary headache disorder, 86% reported improvement of stabbing pains [4]. First-line CH preventives include verapamil and anti-CGRP monoclonal antibodies (e.g., galcanezumab), which have demonstrated efficacy for both CH and comorbid headache features. For acute CH attacks, subcutaneous sumatriptan (6 mg) and high-flow oxygen (100% at 12–15 L/min) remain the gold standards. If stabbing pains persist despite optimal CH control, indomethacin is the most effective agent for refractory PSH [41]. Alternative options include gabapentin, amitriptyline, and melatonin [41]. Clinicians should be vigilant for medication-overuse headache, as patients may overuse acute medications when managing both conditions simultaneously.

### 2.5. Comorbidity of CH with Medication Overuse Headache (MOH)

MOH, defined by ICHD-3 as headache on ≥15 days/month for >3 months due to overuse of acute headache medications, affects ~5% of the general population and is a major cause of chronic refractory headache. While MOH is well-documented in migraine (33% of chronic migraine patients) and tension-type headache [6], its occurrence in CH was long underrecognized [9]. Emerging data show CH patients frequently overuse acute analgesics (e.g., triptans, opioids) to manage severe attacks, predisposing them to MOH—especially those with chronic CH or comorbid migraine/family history of migraine [42].

Epidemiological characteristics. The prevalence of MOH in CH has historically been underrecognized [42]. A large cross-sectional cohort study by Lund et al. (2025) found that among 433 CH participants, 21% met ICHD-3 criteria for medication overuse, with 16% fulfilling criteria for probable MOH. Isolated triptan overuse was uncommon; instead, simple analgesics (52.2%), opioids (29.9%), and combination therapies (20.9%) were most frequently overused [9]. In contrast, an earlier retrospective series by Paemeleire et al. (2006) reported that among 17 CH patients (10 chronic, 7 episodic) with MOH, triptans (*n* = 14) were most commonly overused, followed by opioids (*n* = 10) and simple analgesics (*n* = 9) [42]. Chronic CH (odds ratio 11.4) and comorbid migraine (odds ratio 2.35) were identified as significant associated factors in both studies [9]. Regarding the clinical phenotype of MOH in CH patients, two distinct patterns were observed. Approximately half of the patients presented with a bilateral, dull, featureless daily headache. In contrast, the remaining patients exhibited MOH with at least one migrainous feature, most commonly nausea (35.3%), exacerbation with head movement (29.4%), or a throbbing character of the pain (29.4%) [42].

Potential pathophysiological mechanisms. The pathophysiology of MOH in CH likely involves central sensitization and shared vulnerability with migraine. A personal or family history of migraine was present in 15 of 17 CH-MOH patients reported by Paemeleire et al. (2006), suggesting a genetic or biological predisposition [42]. In contrast to migraine, Centonze et al. (2000) observed that episodic CH patients overusing subcutaneous sumatriptan did not develop tolerance, tachyphylaxis, or rebound syndromes, indicating that the biological response to triptan overuse may differ between the two disorders [43].

Therapeutic implications. Management of MOH in CH requires careful clinical judgment. Medication withdrawal was attempted and successful in 13 of 17 patients in Paemeleire et al.’s (2006) study, supporting withdrawal as an effective strategy [42]. However, Lund et al. (2025) caution against discontinuing triptans when MOH is suspected due to ethical concerns, given the severe disability of CH attacks. Patients with probable MOH showed significantly reduced efficacy of both acute (20.0% vs. 55.9%) and preventive (13.3% vs. 37.3%) treatments compared to those without MOH [9], emphasizing the clinical impact of this comorbidity. Close monitoring is recommended, particularly for patients with chronic CH or comorbid migraine, and future prospective studies are needed to establish definitive withdrawal protocols.

### 2.6. Overlap of CH with Primary Cough Headache (PCH)

According to ICHD-3, PCH is a rare disorder characterized by sudden-onset head pain triggered exclusively by coughing, straining, or other Valsalva maneuvers (but not by prolonged exercise), lasting between 1 s and 2 h, typically bilateral and occipital in location, with neuroimaging mandatory to exclude secondary causes such as Chiari malformation. Some case reports may document the overlap between CH and primary cough headache. Perini and Toso (1998) described a 57-year-old man with unilateral, cough-precipitated attacks exhibiting restlessness and temporal clustering—features of CH [44]. Ko and Rozen (2002) first proposed “Valsalva-induced CH” in a patient whose attacks were triggered solely by coughing/sneezing and prevented by indomethacin [45]. McGeeney (2006) independently confirmed this entity, demonstrating reproducible Valsalva-triggered CH without spontaneous attacks [46]. Together, these cases establish Valsalva-induced CH as a legitimate overlapping phenotype bridging cough headache and classic CH.

### 2.7. Possible Overlap of CH with Primary Headache Associated with Sexual Activity (HSA)

Despite distinct phenotypes, CH and HSA exhibit bidirectional interaction. Sexual activity can trigger CH attacks or, conversely, abort ongoing attacks via hypothalamic modulation [47]. Observational data show 37% of CH patients report improvement with sexual activity, while 50% report worsening [48]. This complex relationship suggests shared hypothalamic circuitry, though formal diagnostic overlap meeting ICHD-3 criteria for both disorders remains to be established.

### 2.8. Possible Overlap of CH with Primary Thunderclap Headache (PTH)

TCH is defined by ICHD-3 as a severe headache of abrupt onset reaching maximum intensity within one minute [49]. While PTH is classified among “other primary headaches” and CH belongs to trigeminal autonomic cephalalgias, their possible overlap is documented in the literature. CH may rarely present with thunderclap onset—a phenomenon documented in clinical series [50]. However, isolated primary and secondary thunderclap headache cannot be clinically differentiated; therefore, any CH patient presenting with thunderclap-onset attack requires urgent neuroimaging to exclude subarachnoid hemorrhage or reversible cerebral vasoconstriction syndrome before a diagnosis of PTH can be established.

### 2.9. Possible Overlap of CH with External-Pressure Headache (EPH)

EPH, classified under ICHD-3, is a primary headache triggered by sustained mechanical compression or traction on the scalp (e.g., from helmets, goggles, or traction devices), with pain maximal at the stimulus site and resolving within one hour after relief. While EPH is well-defined, there is no specific literature on its comorbidity or overlap with CH, and no reported cases document both conditions in the same patient. However, patients with CH exhibit significantly lower pressure pain thresholds and widespread mechanical hyperalgesia, indicating central sensitization. This heightened sensitivity could theoretically predispose them to external-pressure triggers, though empirical evidence is lacking. Moreover, cases of mechanical precipitation of headache attacks have been documented in other trigeminal autonomic cephalalgias, such as CPH, where head flexion or rotation can trigger attacks [51], suggesting that mechanical triggers may be relevant across this headache spectrum. Nevertheless, formal overlap between CH and EPH remains unsubstantiated.

## 3. Conclusions

CH frequently co-occurs with several other primary headache disorders, most commonly migraine, followed by other TACs, PSH, and MOH. Overlapping phenotypes—such as cluster-migraine and Valsalva-induced CH—suggest shared pathophysiological mechanisms involving CGRP signaling, hypothalamic dysfunction, and trigeminal sensitization. However, formal overlap with tension-type headache, hypnic headache, or external-pressure headache has yet to be substantiated by well-documented case reports.

Recognizing these comorbidities in CH is clinically significant for several reasons. First, the presence of a second headache disorder may alter the overall symptom profile, potentially delaying or obscuring the correct diagnosis. For example, co-occurring migraine with aura can mask the typical CH presentation. Second, comorbid headache disorders can influence treatment response. While patients with pure CH often respond well to acute therapies such as subcutaneous sumatriptan or high-flow oxygen, the coexistence of another headache phenotype may necessitate a broader, individualized therapeutic strategy. Third, the cumulative burden of multiple headache disorders may substantially impair quality of life beyond that attributed to CH alone.

In summary, CH comorbidity with other headache disorders is clinically relevant, often underrecognized, and warrants systematic prospective investigation to improve diagnostic accuracy and treatment outcomes.

## Figures and Tables

**Table 1:** Prevalence of comorbid primary headache disorders in patients with cluster headache.

Comorbid Condition	Prevalence in CH Cohorts	Key Source/Study Design
Migraine	13.6–36%	Multiple cohort studies; Norwegian registry data show 9.8% in men, 23.1% in women [7,8]
TTH	Underrecognized; not formally established	No well-documented prevalence data; interictal “shadow pain” may obscure diagnosis
Other TACs	Rare; documented in case reports and small series	Sequential/concurrent occurrence reported but formal comorbidity rates not established
PSH	2–33%	Literature synthesis; PSH reported in 10% of a tertiary PSH cohort with comorbid CH [4]
MOH	16% (probable MOH); 21% (medication overuse)	Lund et al. (2025), Cephalalgia, *n* = 433; chronic CH (OR 11.4) is a significant associated factor [9]
PCH	Case reports only	“Valsalva-induced CH” phenotype documented but no systematic prevalence data
HSA	No formal comorbidity data	Bidirectional interaction described; 37% report improvement, 50% worsening with sexual activity
PTH	Case reports only	CH may rarely present with thunderclap onset; requires urgent neuroimaging to exclude secondary causes
EPH	No documented comorbidity	Theoretically plausible due to central sensitization, but no empirical evidence

## Data Availability

No new data were created or analyzed in this study. Data sharing is not applicable to this article.

## Abbreviations

The following abbreviations are used throughout this manuscript: CH, cluster headache; TACs, trigeminal autonomic cephalalgias; ICHD-3, International Classification of Headache Disorders, 3rd edition; TTH, tension-type headache; PSH, primary stabbing headache; MOH, medication-overuse headache; PH, paroxysmal hemicrania; HC, hemicrania continua; SUNCT, short-lasting unilateral neuralgiform headache attacks with conjunctival injection and tearing; SUNA, short-lasting unilateral neuralgiform headache attacks with cranial autonomic symptoms; PCH, primary cough headache; HSA, headache associated with sexual activity; PTH, primary thunderclap headache; EPH, external-pressure headache; CGRP, calcitonin gene-related peptide; CPH, chronic paroxysmal hemicrania; SUNHA, short-lasting unilateral neuralgiform headache attacks; MRI, magnetic resonance imaging.
